# Cognitive Performance and Cerebrospinal Fluid Biomarkers of Neurodegeneration: A Study of Patients with Bipolar Disorder and Healthy Controls

**DOI:** 10.1371/journal.pone.0127100

**Published:** 2015-05-08

**Authors:** Sindre Rolstad, Joel Jakobsson, Carl Sellgren, Carl-Johan Ekman, Kaj Blennow, Henrik Zetterberg, Erik Pålsson, Mikael Landén

**Affiliations:** 1 Institute of neuroscience and physiology, the Sahlgrenska Academy at the Gothenburg University, Gothenburg, Sweden; 2 Department of Medical Epidemiology and Biostatistics, Karolinska Institutet, Stockholm, Sweden; 3 Department of Clinical Neuroscience, Karolinska Institutet, Stockholm, Sweden; 4 UCL Institute of Neurology, Queen Square, University College London, London, United Kingdom; UW Medicine Neuropathology, UNITED STATES

## Abstract

The purpose of the present study was to investigate if cerebrospinal fluid (CSF) biomarkers of neurodegeneration are associated with cognition in bipolar disorder and healthy controls, respectively. CSF concentrations of total and phosphorylated tau, amyloid beta (Aβ)1-42, ratios of Aβ42/40 and Aβ42/38, soluble amyloid precursor protein α and β, and neurofilament light chain protein were analyzed in relation to neuropsychological performance in 82 euthymic bipolar disorder patients and 71 healthy controls. Linear regression models were applied to account for performance in five cognitive domains using the CSF biomarkers. In patients, the CSF biomarkers explained a significant proportion of the variance (15–36%, *p*=.002 - <.0005) in all cognitive domains independently of age, medication, disease status, and bipolar subtype I or II. However, the CSF biomarkers specifically mirroring Alzheimer-type brain changes, i.e., P-tau and Aβ1-42, did not contribute significantly. In healthy controls, CSF biomarkers did not explain the variance in cognitive performance. Selected CSF biomarkers of neurodegenerative processes accounted for cognitive performance in persons with bipolar disorder, but not for healthy controls. Specifically, the ratios of Aβ42/40 and Aβ42/38 were consistently associated with altered cognitive performance.

## Introduction

The hallmark of bipolar disorder is recurrent episodes of depression and mania or hypomania [1]. The worldwide prevalence of the main subtypes of bipolar disorder, type I and II, is estimated to about 1–3% [2]. The disorder is associated with high societal costs, of which indirect costs due to sick leave and early retirement are the main drivers [3]. It has been increasingly recognized that cognitive dysfunction is an important predictor of functional outcomes in bipolar disorder [4].

Meta-analyses suggest that attention/speed, memory, and executive functions are impaired in euthymic bipolar disorder [5, 6]. An independent individual patient data meta-analysis provided further evidence of significant cognitive impairment in bipolar disorder, albeit less substantial than previous reports suggested [7]. Another meta-analysis found that differences between bipolar type I and II are negligible with the exception of memory and semantic fluency [8]. Whereas there is some evidence of cognitive deterioration during the course of illness [7, 9], most cognitive functions appear to remain persistently impaired over time [10].

No biological correlates of cognitive impairment in bipolar disorder have been established.

Meta-analyses of structural magnetic resonance imaging (MRI) studies report morphological differences between persons with bipolar disorder and controls [11, 12], but these structural abnormalities have not been linked to cognitive deficits [13]. It is hence undecided whether the neuroimaging findings in bipolar disorder indicate a neurodegenerative process, a premorbid condition, effects of alcohol intake, altered hormone levels, or medication effects [14].

In two recent studies, we investigated the applicability of cerebrospinal fluid (CSF) biomarkers to study neurodegenerative processes in bipolar disorder [15, 16]. We found decreased concentrations of the soluble forms amyloid precursor protein (APP)—sAPPα and sAPPβ - and higher ratios of amyloid β (Aβ) 42/40 and Aβ42/38 in persons with bipolar disorder compared with healthy controls [16]. The physiological role of APP is not fully understood, but it has been linked to synaptic formation and repair as well as axonal regeneration [17]. APP has also been suggested to be important for neural connectivity, plasticity, and activity, as well as for memory functions. We found no group difference between bipolar disorder patients and controls with respect to total or phosphorylated tau (T-tau/P-tau) that reflect axonal damage and neurofibrillary degeneration [18], or Aβ1–42 that indicate plaque deposition [19]. In the subsequent study, however, we found higher mean CSF concentrations of neurofilament light chain protein (NFL) in persons with bipolar disorder compared to controls [15]. NFL is a cytoskeletal constituent of intermediate filaments. Increased CSF NFL is considered to reflect neuronal and axonal degeneration and loss [20]. Taken together, these previous studies suggested that altered APP metabolism and axonal injury might occur in bipolar disorder.

CSF biomarkers of neurodegeneration have been linked to cognitive impairment in other disorders [18, 21]. It is hence not farfetched to suggest that they might also be associated with cognitive dysfunction in bipolar disorder. Clarifying this issue is important for at least two reasons. First, it might yield insights as to the biological underpinnings of cognitive impairment in bipolar disorder, which in turn is important for identifying treatment targets to alleviate cognitive impairment. Second, biomarkers of neurodegeneration might prove useful to predict worsening of cognitive function during the course of illness. If biomarkers could help identifying vulnerable individuals, targeted intervention programs to prevent cognitive decline could be developed.

The aim of this study was to evaluate potential associations between CSF biomarkers of degeneration (T-tau, P-tau, Aβ1–42, Aβ42/40 and Aβ40/38 ratios, sAPPα and sAPPβ, and NFL) and cognitive function in patients with bipolar disorder. Regression models with five aggregated cognitive domains were applied using CSF biomarkers as predictors and covariates as appropriate. The models were repeated in healthy age- and sex-matched controls to determine if observed associations were disease dependent.

## Methods

### Patients

The St. Göran Bipolar Project is a clinical longitudinal study of persons with bipolar disorder. The procedures in this project have been described in detail elsewhere [22]. In brief, patients were enrolled at the bipolar outpatient unit at the Northern Stockholm Psychiatric Clinic (Stockholm, Sweden). The inclusion criteria for the St. Göran Bipolar Project are ≥18 years of age, fulfilling the DSM-IV criteria for bipolar disorder type I or II. Exclusion criteria were inability to complete the standard clinical assessment or incapability of providing informed consent.

The key clinical assessment instrument was a Swedish version of the Affective Disorder Evaluation ADE), which is a standardized interview protocol developed for the Systematic treatment Enhancement Program of Bipolar Disorder (STEP-BD) [23]. The ADE directs the interviewer through a systematic assessment of the patient’s current mental state, psychiatric history, and diagnosis according to DSM-IV criteria as per the Structured Clinical Interview for DSM-IV (SCID) [24]. The ADE includes a social anamnesis, and a medical history. The lifetime severity of bipolar disorder is rated using the 7-point Likert scale Clinical Global Impression (CGI), which ranges from healthy to extremely ill. In addition to the ADE, the Mini International Neuropsychiatric Interview (M.I.N.I.) [25] was completed to screen for other psychiatric diagnoses than bipolar disorder. Alcohol Use Disorders Identification Test (AUDIT) and the Drug Use Disorders Identification Test (DUDIT) were used to screen for substance and alcohol abuse, as well as serum levels of carbohydrate-deficient transferrin [26]. The ADE and M.I.N.I. interviews were conducted by board-certified psychiatrists working at the tertiary bipolar outpatient unit, or residents in psychiatry completing their training at this unit. To minimize risk of inter-rater bias, a best-estimate diagnostic decision was made based on all information available at admission by a consensus panel of experienced board certified psychiatrists specialized in bipolar disorder. All available sources of information, encompassing patient interview, case records and, if available, interview with the next of kin, were utilized in the diagnostic assessment.

Both the CSF sampling and the cognitive examination procedures were carried out when patients were in a euthymic mood. Euthymia was defined as MADRS (Montgomery–Åsberg Depression Rating Scale) and YMRS (Young Mania Rating Scale) scores <14.

### Controls

Controls were included to evaluate the extent to which CSF biomarkers play a specific role in bipolar disorder. Age- and sex-matched healthy, population-based controls were randomly selected by Statistics Sweden and contacted by mail. Given an expected response rate of 1:7, seven invitations were sent out per enrolled patient. Fourteen percent of the invited controls responded to the invitation to participate, and were subjected to a preliminary telephone screening to exclude severe mental health, neurological problems, and substance abuse. Thus, 75 individuals were excluded due to drug use, no longer willing to participate, or somatic illness. Eligible persons were scheduled for a personal examination and investigated to exclude mental illness by a psychiatrist using the M.I.N.I. and selected parts of the ADE. The control subjects underwent blood-sampling, lumbar puncture, neuropsychological testing and self-rating scales. The procedures were identical in patients and controls, except that controls completed all investigations during the same day, whereas CSF sampling and the neuropsychological testing occurred at separate occasions for patients. Controls presenting potentially pathological findings were discussed between examining clinician, primary investigator, and study coordinator at case conferences. Exclusion criteria were: any current psychiatric disorder, a family history of schizophrenia or bipolar disorder in first-degree relatives, drug or alcohol abuse (based on DUDIT and AUDIT as well as serum levels of carbohydrate-deficient transferrin), neurological conditions except mild migraines, pregnancy, untreated endocrine disorders, dementia, and severe personality disorder.

### Ethics

The study was approved by the Regional Ethics Committee in Stockholm (case no. 2005/554-31/3) and conducted in accordance with the latest Helsinki Protocol. After complete description of the study, all enrolled patients and controls consented orally and in writing to participate in the study.

### CSF sampling and biomarker analyses

To reduce the risk of diurnal fluctuations, lumbar puncture was performed at 0900–1000 AM after night fasting. The spinal needle was inserted into the L3/L4 or L4/L5 interspace, and a total volume of 12 ml of CSF was drawn, gently inverted to avoid gradient effects, and divided into 1.0–1.6 ml aliquots that were stored at -80°C pending analysis. For ethical reasons, patients were not taken off their prescribed medication at the time of the sampling. All samples were thawed and refrozen once before analysis. All biochemical analyses were performed at the Clinical Neurochemistry Laboratory in Mölndal, Sweden, by board-certified laboratory technicians blinded with respect to clinical information. The CSF concentrations of sAPP α and β were determined using the MSD sAPPα /sAPPβ Multiplex Assay, while CSF Aβ38, Aβ40, and Aβ42 were analyzed using the MSD Human/Rodent (4G8) Assay (Meso Scale Discovery, Gaithersburg, MD, USA), as described previously [27]. CSF concentrations of hyperphosphorylated Tau (P-tau), total tau (T-tau), and Aβ1–42 were measured simultaneously by the Luminex xMAP technology using the Inno-Bia AlzBio3 kit (Innogenetics, Zwijndrecht, Belgium), as described preciously [28]. NFL was analyzed as previously described with a commercial ELISA assay (NF-light, UmanDiagnostics AB, Umeå, Sweden). The intra-assay coefficient of variability was <10% for all biomarkers and the inter-assay coefficient of variability varied from 2% (Aβ38) to 20% (sAPPβ).

### Neuropsychological examination

The administered neuropsychological test battery accords the recent recommendations from the International Society for Bipolar Disorders [29] covering cognitive domains deemed important for characterizing cognition in bipolar disorder. In order to approximate a complete picture of the participants’ cognitive status, several aspects of function were assessed within each domain. Verbal tests were mixed with nonverbal in each session and the sequence of tests was administered in a way that reduced the risk of contamination on the memory tests. Licensed psychologists administered all tests to the patients whereas trained psychology students under supervision by a licensed psychologist administered tests to the healthy controls. In general, two sessions were required for patients and one for controls. Scores on the neuropsychological tests were used to create summary indices of the specific cognitive domains guided by common measurement properties and reference literature [30, 31]. In order to combine the scores from different tests, we converted the raw scores on each of the tests to z-scores using the mean and SD of the healthy controls and averaged the z-scores of the tests within a given cognitive domain to yield a domain score. This procedure was applied for patients as well as controls. Specific neuropsychological tests and functions measured are displayed in Table 1. The purpose of analyzing cognitive domains rather than individual cognitive tests is to succinctly communicate the underlying measuring entities [32], decrease the test-specific associations, and to reduce the potential alpha inflation resulting from a larger battery of tests.

**Table 1:** Cognitive domains and functions assessed in the St Göran bipolar project.

Cognitive domain	*Specific functions* and neuropsychological tests (battery)
Speed and attention	*Cognitive speed*: Trail making Test number sequencing (D-KEFS); Digit Symbol-Coding (WAIS-III)
Learning and memory	*Verbal episodic memory*: Claeson-Dahl; *Non-verbal episodic memory*: Rey Complex Figure recall; *Working memory*: Digit Span (WAIS-III), Letter-Number Sequencing (WAIS-III)
Visuospatial functions	*Spatial organization*: Rey Complex Figure copy; *Construction*: Block Design (WAIS-III)
Verbal functions	*Abstraction*: Similarities (WAIS-III); *Verbal fluency*: Verbal Fluency Test (D-KEFS)
Executive functions	*Inhibition*: *Color-Word interference* (D-KEFS), Design Fluency (D-KEFS), Trail Making Test number-letter sequencing (D-KEFS), Tower Test (D-KEFS)*; Distractibility*: Continuous Performance Test

WAIS-III = Wechsler’s Adult Intelligence Scale version III, D-KEFS = Delis-Kaplan executive function system.

### Statistical analysis

The primary outcome of this study was the ability of the CSF biomarkers to explain cognitive performance in bipolar disorder. The applicability of the biomarkers to account for cognitive performance was further tested in the healthy age- and sex-matched controls.

Preliminary analyses were performed to ensure no violation of the following prerequisites for regression: linearity, normality, absence of multicollinearity, and homoscedasticity. Variables that violated requirements for linearity were transformed as appropriate and variables that were highly inter-correlated were excluded for the model of interest. Linear regression was performed to assess the degree to which CSF biomarkers can explain the variance in the aggregated cognitive domain scores. CSF biomarkers were entered as independent variables in all models. The following variables were included as covariates for patients: age, sex, bipolar subtype, CGI, MADRS, YMRS, treatment with any mood stabilizer, lithium, anticonvulsants, antidepressants, antipsychotics, benzodiazepines, and anxiolytics (non-benzodiazepine anti-anxiety medication). For healthy controls, age and sex were the only applicable covariate.

We report the adjusted *r* ^2^ of the model, the standardized beta values, and the *p*-value (two-tailed tests) of the individual variables. Analysis of variance and chi-square, where applicable, were used for group comparisons. P-values <.05 were considered significant. Alpha correction was not applied. To test if the covariates accounted for the influence of the CSF biomarkers on cognition, mediation effects were assessed using bootstrapping for continuous mediators and logistic mediation analysis for dichotomous mediators (medication). Analyses were performed using SPSS version 21 (Armonk, NY: IBM Corp.) and SAS JMP version 10 (Cary, NC: SAS inst.).

## Results

The current study included 82 cases of bipolar disorder type I and II that had completed sampling of CSF and cognitive examination (Table 2). The patient sample was predominantly female (58.5%) with a mean (SD) age of 38.4 (±12.6) and an average of 12.9 (±2.8) years of education. The mean (SD) CGI score was 4.47 (±. 98). Most patients were bipolar type I and a majority were prescribed mood stabilizers such as lithium, valproate, or lamotrigine (86.6%, 61% lithium in total) at the time of examination. To investigate if the associations between CSF biomarkers and cognitive performance were specific to bipolar disorder, this study also included healthy controls (Table 2).

**Table 2:** Demographics and clinical characteristics of patients with bipolar disorder type I and II and healthy controls.

	Patients (N = 82)		Controls (N = 71)	

	Mean/*frequency*	SD/*%*	Mean/*frequency*	SD/*%*
Females	*48*	*58*.*5*	*44*	*61*.*9*
Age	38.3	12.5	37.8	14.6
Education, years	12.9	2.8	14.1	1.7
Bipolar type I/II	*53/29*	*64*.*6/35*.*4*	-	-
Clinical Global Impression	4.4	.9	-	-
Mood stabilizers	*71*	*86*.*6*	-	-
Antidepressants	*34*	*41*.*5*	-	-
Lithium	*50*	*61*	-	-
Anxiolytics	*20*	*24*.*4*	-	-
Benzodiazepines	*23*	*28*	-	-
Anticonvulsants	*36*	*43*.*9*	-	-
Antipsychotics	*17*	*20*.*7*	-	-
sAPPα^a^	723.26	(307.81)	859.58	(282.80)
sAPPβ^b^	300.29	(156.87)	354.93	(152.88)
Aβ42/40^c^	.1152	(.0199)	.1107	(.0177)
Aβ42/38^d^	.7729	(.1133)	.7345	(.1103)
Aβ1–42^e^	253.88	(62.33)	254.38	(55.42)
T-tau^f^	34.32	(12.48)	37.17	(14.09)
P-tau^g^	26.72	(6.79)	28.27	(6.89)
NFL^h^	485.73	(425.62)	254.38	(55.42)

Frequencies and percentages are italicized.

^a^sAPPα/^b^sAPPβ (ng/ml) = secreted form of beta-amyloid precursor protein α/β;

^c^Aβ42/40 = CSF amyloid beta 42/40 ratio (pg/ml);

^d^Aβ42/38 = CSF amyloid beta 42/38 ratio (pg/ml);

^e^Aβ1–42 = CSF amyloid beta 1–42 (pg/ml);

^f^T-tau = CSF total tau (pg/ml);

^g^p-tau = phosphorylated tau (pg/ml);

^h^NFL = Neurofilament light subunit (pg/ml)

Data from the St. Göran cohort on test specific cognitive performance [33] as well as CSF biomarkers concentrations have been presented previously [15, 16]. However, in the present study, only subjects that had completed both cognitive testing and lumbar puncture were included. In the present study, controls did not differ significantly from patients with respect to age and gender distribution, but had significantly more years of education (*F* (1,151) = 8.7, *p* =. 004). Patients’ sAPPα (*F* (1,151) = 8.1, *p* =. 005) and sAPPβ (*F* (1,151) = 4.7, *p* =. 03) were lower whereas their Aβ42/38 ratio (*F* (1,151) = 4.4, *p* =. 03) and NFL concentrations (*F* (1,151) = 5.4, *p* =. 02) were higher. Patients performed worse than healthy controls in the domains of memory (*F* (1,151) = 13.6, *p* <.0005) and verbal functions (*F* (1,151) = 7.1, *p* =. 009) (Table 3).

**Table 3:** Cognitive performance for patients with bipolar disorder and healthy controls.

	Patients	Controls
	Mean (SD)	Mean (SD)
Memory functions	-.26 (.46)	.00 (.39)
Executive functions	-.62 (.69)	-.42 (.66)
Visuospatial functions	-.23 (1.01)	.03 (.73)
Speed/attention	.09 (.75)	-.09 (.50)
Verbal functions	-.27 (.76)	.02 (.59)

### Memory/learning domain

The CSF biomarkers explained a significant proportion of the variance in the memory domain; the model as a whole accounted for 15% (adj. r^2^ =. 15, F(4, 82) = 5.0. *p*. 001) of the observed variance (Table 4). Higher concentrations of NFL and a higher ratio of Aβ42/40 were associated with worse memory performance, whereas a higher ratio of Aβ42/38 was associated with better memory performance. NFL was the most influential predictor in this model (beta = -.37) indicating that an increase of 425 pg/ml NFL (1 SD) reduced memory performance by-.37 SD.

**Table 4:** Cerebrospinal fluid biomarkers associated with cognitive performance in bipolar disorder.

Dependent variable	Predictor	Beta	T	*P*
Memory	NFL^a^	-.37	-3.64	<.0005
	Aβ42/40^b^	-.30	-2.58	.01
	Aβ42/38^c^	.27	2.60	.01
Attention/speed	Antipsychotics	.35	-2.19	.03
	Benzodiazepines	-.31	-2.15	.03
	sAPPα^d^	-.27	-2.22	.03
	Age	-.23	-2.40	.01
	Anticonvulsants	.21	2.09	.04
	Aβ42/40	-.07	-2.47	.01
	Aβ42/38	.06	2.34	.02
Executive functions	Benzodiazepines	-.69	-2.63	.01
	Anxiolytics	.61	2.72	.008
	T-tau	-.35	3.73	<.005
Verbal functions	NFL	-.39	-3.76	<.005
	Aβ42/40	-.18	-2.07	.04
	Anticonvulsants	.14	3.11	.002
	T-tau	.14	2.11	.003
Visuospatial functions	Age	-.60	-6.09	<.005
	Aβ42/40	-.52	3.30	<.005
	Aβ42/38	.48	2.27	.04
	T-tau	.33	2.63	.01
	Lithium	.17	2.10	.03

Non-significant results are not displayed.

^a^NFL = Neurofilament light subunit (pg/ml);

^b^Aβ42/40 = CSF amyloid beta 42/40 ratio (pg/ml);

^c^Aβ42/38 = CSF amyloid beta 42/38 ratio (pg/ml);

^d^sAPPα (ng/ml) = secreted form of beta-amyloid precursor protein;

^e^T-tau = CSF total tau (pg/ml)

As working memory can be conceived as a separate memory system [34], the association between CSF biomarkers and memory/learning performance was repeated without the working memory tests (Digit Span and Letter-Number Sequencing). Whereas a smaller percentage was accounted for when working memory was removed from the domain (adj. r^2^ =. 12, F(4, 82) = 4.4. *p* =. 006), the contribution of the CSF biomarkers NFL, Aβ42/40, and Aβ42/38 on memory remained essentially unchanged (data not shown). We also performed a separate analysis of the association between working memory and CSF biomarkers and found that the CSF biomarkers explained a significant proportion of the variance in working memory performance (adj. r^2^ =. 27, F(4, 82) = 8.0. <.0001). Low working memory performance was associated with an elevated concentration of NFL (beta = -.43, *p*<.0001) and a higher Aβ42/38 ratio (beta = -.28, *p* =. 03). Use of lithium was associated with increased working memory performance (beta =. 27, *p =*. 001).

### Attention/speed domain

In total, the model explained 24% (adj. r^2^ =. 24, F(8, 75) = 4.01. *p* <.0005) of the variance in speed and attention performance. Low performance in the attention/speed domain was associated with lower concentrations of sAPPα and a lower Aβ42/40 ratio, whereas a higher Aβ42/38 ratio again was associated with higher performance. Lower age and use of anticonvulsants and antipsychotics were positively associated with speed and attention, whereas use of benzodiazepines had a negative impact. sAPPα was the only CSF biomarker with a marked impact on attention/speed (beta = -.27, lower concentration was associated with worse performance). Use of benzodiazepines was negatively associated with attention (beta = -.31).

### Executive domain

The model accounted for a significant proportion (29%, adj. r2 =. 29, F(9, 75) = 4.3. p <.0005) of the executive domain scores. Higher concentrations of T-tau explained reduced executive performance (beta = -.35). Use of anxiolytics (except benzodiazepines) was associated with increased performance, whereas benzodiazepine use was associated with reduced performance.

### Verbal domain

In total, CSF biomarkers and covariates accounted for 21% (adj. r^2^ =. 21, F(10, 76) = 3.08. *p* =. 002) of the variance in performance in this domain. Better verbal performance was associated with higher T-tau concentrations and a lower Aβ42/40 ratio. Higher concentrations of NFL was most strongly negatively associated with verbal performance (beta = -.39). Use of anticonvulsants was negatively associated with verbal functioning.

### Visuospatial domain

**Fig 1:**
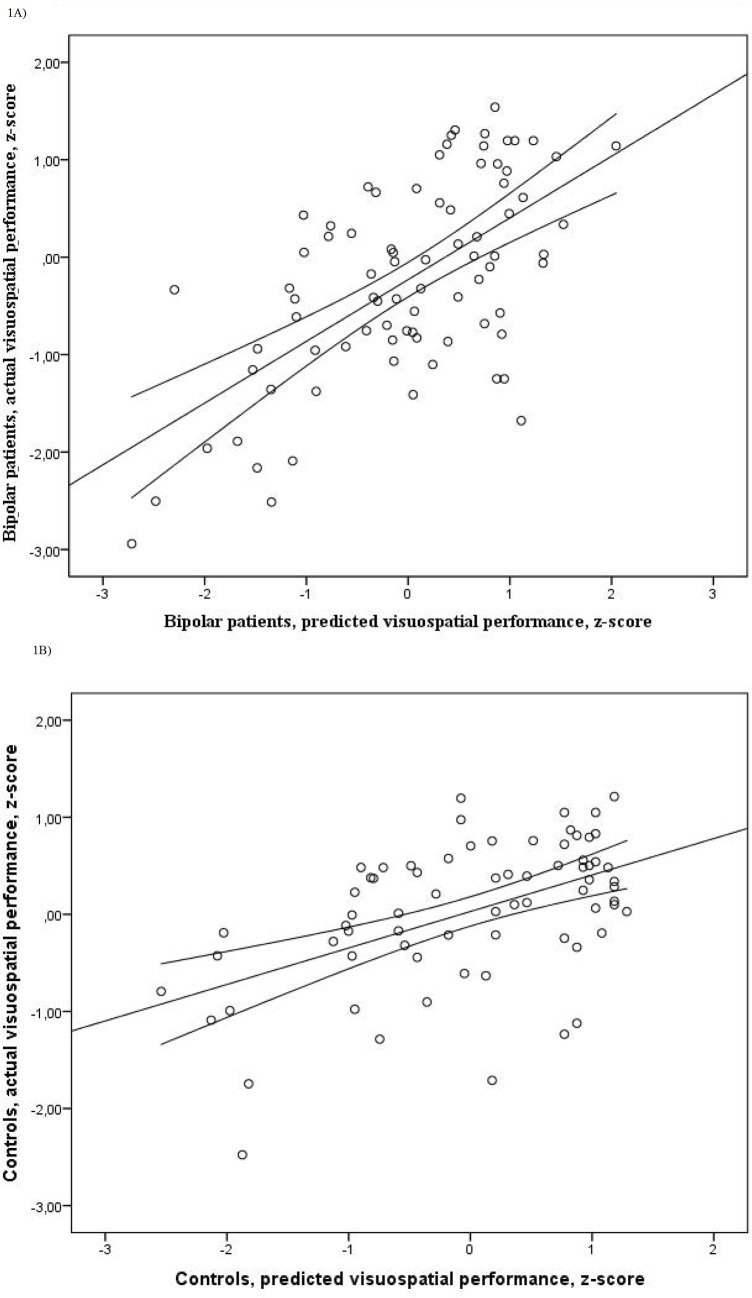
Association between actual and predicted visuospatial score in bipolar patients and healthy controls. Figures display actual and predicted visuospatial performance in z-scores in bipolar disorder (1A) and healthy controls (1B). In patients with bipolar disorder cerebrospinal fluid (CSF) Amyloid β (Aβ) 42/40 ratio, CSF Aβ42/38 ratio, CSF total tau, age, and lithium contributed significantly to the model (adj. r^2^ =. 36, *p* = <.0005). In patients with bipolar disorder only sex and age were associated with visuospatial functions (adj. r^2^ =. 23, *p* = <.0005).

The total variance explained in visuospatial performance was 36% (adj. r^2^ =. 36, F(11, 82) = 5.37. *p* = <.0005) (Fig 1A). Higher concentrations of T-tau, higher Aβ42/38 ratio, and lower Aβ42/40 ratio were associated with increased visuospatial performance. Use of lithium and lower age was positively associated with visuospatial functions. Age (beta = -.60), Aβ42/40- (beta = -.52) and Aβ42/38 (beta =. 48) ratios were the most influential predictors in explaining visuospatial performance.

### Mediation effects

Mediation effects were not found for any of the covariates included in the regression models (results not displayed); CSF biomarkers and covariates contributed independently to the variance observed in the cognitive domains.

### Controls

CSF biomarkers did not account for any variance in cognitive performance for the controls. The only significant predictors of cognitive performance in healthy controls were sex and age for visuospatial functions (Fig 1B) and sex for verbal functions (Table 5).

**Table 5:** Variables associated with cognitive performance in healthy controls.

Dependent variable	Predictor	Beta	T	*P*
Verbal functions	Sex	-.44	-3.93	<.0005
Visuospatial functions	Sex	-.28	-2.60	.01
	Age	-.38	-3.60	.001

Non-significant results are not displayed.

## Discussion

This is the first study investigating the relationship between CSF biomarkers of neurodegeneration and cognition in bipolar disorder. The biomarkers independently accounted for a significant part of the individual variability of cognitive performance in the bipolar disorder group. Intriguingly, the findings could not be reproduced in the control sample. This suggests that an association with CSF neurodegeneration biomarkers and cognitive performance is not a general phenomenon. It remains to be elucidated if these associations are specific to persons with bipolar disorder, or if CSF biomarkers of neurodegeneration might also be associated with cognitive performance in other psychiatric disorders such as schizophrenia.

Biomarkers and covariates best explained the variance in visuospatial functions, which is somewhat surprising as visuospatial functions are typically relatively well preserved in bipolar disorder [7]. As the individual tests constituting the visuospatial domain—Rey Complex Figure Test and Block Design—also load on executive functions, there is a possibility that some of the variance in this domain may be accounted for by other cognitive functions [35]. The relatively large proportion of variance accounted for by CSF biomarkers in the verbal domain was also unexpected as most studies have reported intact verbal functions in bipolar disorder [6]. By contrast, attention and processing speed was only weakly accounted for by the biomarkers; covariates had a stronger influence on performance.

In the group with bipolar disorder, higher concentration of NFL, as well as higher Aβ42/40 ratio and lower Aβ42/38 ratio, was consistently associated with decreased cognitive performance. Altered ratios of Aβ were the CSF biomarkers that accounted for most of the variance in verbal performance. Again, this finding could be related to cross-loadings on executive functions. NFL was the most important predictor of memory performance. However, the model only accounted for 15 percent of the variance in the memory domain, which dropped to 12 percent when working memory tests were removed from the memory/learning domain. Working memory alone was more strongly associated with CSF biomarkers (27%) and NFL was the CSF biomarker that contributed the most to the model. Nonetheless, this suggests that neuronal and axonal dysfunction or degeneration might contribute to reduced memory performance in bipolar disorder. Aβ40 is more adhesive than the Aβ38 isoform and thus considered more harmful [36]. A decrement in both ratios (42/40 and 42/38) would normally be associated with reduced neuropsychological performance [37]. Our finding that a *higher* Aβ42/40 ratio was associated with decreased cognitive performance is therefore somewhat puzzling. However, the Aβ42/38 and Aβ42/40 ratios have also been found to increase prior to deposition of plaque in Alzheimer’s disease [38]. The association between increased Aβ42/40 ratio and cognitive impairment could thus be interpreted as a response to neurotoxicity. Alternatively, elevated Aβ ratios can indicate a γ-secretase dysfunction. γ-secretase cleaves amino acids 37–40 and 42 of the Aβ domain, and the Aβ ratios are expected to increase if this enzyme is functioning less well. Indeed, impaired γ-secretase has been associated with impaired brain plasticity in animal models [39].

T-tau was associated with cognitive performance in three domains, but inconsistently so; higher T-tau concentrations was positively associated with verbal and visuospatial functions, but negatively associated with executive functions. Moreover, there was no difference in CSF T-tau between patients and controls. Hence, the value of T-tau as a predictor of cognitive performance might be limited. Also, sAPPβ was only associated with performance in one cognitive domain; lower sAPPβ concentrations correlated with decreased speed/attention performance. Furthermore, the more AD specific biomarkers P-tau and Aβ1–42 were not associated with cognitive performance in any cognitive domain. As P-tau and Aβ1–42 are established biomarkers for current and future cognitive performance in mild cognitive impairment and AD [21], findings from the current study accords with the study by Jakobsson and colleagues who found no evidence of an Alzheimer like CSF pattern in patients with bipolar disorder [16]. Although there is some evidence that psychiatric disorders may increase the risk of dementia [40], a neuropathological study found no increase of amyloid plaques or neurofibrillary tangles in post-mortem brain tissues in a sample of psychiatric patients [41]. Hence, it is more likely that the associations between CSF biomarkers and cognitive domains in patients reflect a neurotoxic state rather than a neurodegenerative process. As the mean age of this sample was relatively low (38 years) we cannot rule out the possibility that neurodegenerative processes are present in an older cohort.

A limitation of this study is that sampling of CSF was conducted on average 7 months after completion of the neuropsychological examination. Whereas the delay is not ideal, it is not likely that it has affected the results as neuropsychological performance is stable in bipolar disorder. Also, the studied CSF biomarkers are steady over a period of 2 years in patients with Alzheimer’s disease [42]. Although evidence is lacking it is not farfetched to assume that the CSF biomarkers are also stable in patients with bipolar disorder over a short time span. The lack of correction for alpha inflation is another limitation of this study. As this study only included patients who had undergone lumbar puncture and a neuropsychological examination there is a risk of exclusion bias. Whereas the patients included in the current study did not differ in terms of demographic or clinical characteristics (data not shown), we cannot rule out the possibility that patients with a certain phenotype have been excluded in this study. It is possible that the arrangement of the cognitive tests would have been different if the sample size had permitted confirmatory factor analysis. It is not unlikely that a larger proportion of the cognitive tests would have loaded on executive functions. Whereas several covariates of importance to cognitive processes in bipolar disorder were analyzed, the sample size did not allow for inclusion of all covariates of interest. For example, the potential influence of education on cognitive functioning was not analyzed in the current study. The included patients were euthymic at the time of the investigation, which precludes any speculation as to how CSF biomarkers affect cognition in non-euthymic states. As episodes of mania, hypomania, and depression were not recorded during the investigation it cannot be ruled out that the associations seen in this study are sequelae following mania/depression. There were, however, no significant associations between MADRS/YMRS scores and cognition. Finally, whereas the effect of medication was controlled for, this cross-sectional study cannot rule out that medication influences CSF concentrations.

In conclusion, we found that CSF biomarkers of neurodegeneration were associated with cognitive performance in euthymic bipolar disorder, but not in healthy controls, in all cognitive domains independently of age, medication, disease status, and bipolar subtype. Notably, ratios of Aβ42/40 and Aβ42/38 were consistently associated with altered cognitive performance. It remains to be studied whether CSF biomarkers can also be utilized to predict changes in cognitive functioning during the course of illness.
